# No Association Between Face Recognition and Spatial Navigation: Evidence from Developmental Prosopagnosia and Super-Recognizers

**DOI:** 10.3390/brainsci15111140

**Published:** 2025-10-24

**Authors:** Alejandro J. Estudillo, Olivia Dark, Jan M. Wiener, Sarah Bate

**Affiliations:** School of Psychology, Bournemouth University, Talbot Campus, Poole House, Poole BH12 5BB, UK; aestudillo@bournemouth.ac.uk (A.J.E.); olivia.dark1@outlook.com (O.D.); jwiener@bournemouth.ac.uk (J.M.W.)

**Keywords:** developmental prosopagnosia, face recognition, individual differences, navigation, super-recognizers

## Abstract

**Background/Objectives:** Previous studies have reported associations between prosopagnosia and spatial navigation, but it remains unclear whether this link is merely coincidental (i.e., observable only in prosopagnosia) or genuinely interdependent (i.e., such that variation in one ability predicts variation in the other across the full spectrum of face-recognition abilities). This study aimed to directly test this possibility by examining the relationship between face recognition and navigational skills in developmental prosopagnosics (DPs), super-recognizers (SRs), and control participants. **Methods**: Eighteen DPs, sixteen SRs, and twenty-eight control participants were tested in a recently validated route-learning task, in which they were asked to memorize a route from a first-person perspective. In the subsequent test stages, both route repetition and route retracing were assessed. **Results**: Group analyses showed comparable performance in route repetition and retracing across the three groups. Single-case analyses confirmed these findings and indicated that only two DPs and two SRs performed worse than control participants in route retracing. **Conclusions**: These findings suggest that spatial navigation and face recognition are not directly associated and therefore appear to be different skills.

## 1. Introduction

Face recognition is a high-level cognitive process that enables individuals to interact effectively with others. This complex cognitive process entails a set of several subprocesses, including the perception and categorization of a visual stimulus as a face, the matching of this percept to a memory representation of that face, and the retrieval of semantic information associated with the person [1,2,3]. Despite its importance, face identification shows substantial variability across individuals [4,5,6,7,8,9]. For example, this ability can be lost due to brain damage, as in the case of acquired prosopagnosia (AP) [10,11,12,13], or it may be absent from birth, as seen in developmental prosopagnosia (DP) [14,15,16,17,18]. At the other end of the spectrum, super-recognizers (SRs) possess exceptional face recognition abilities, to the extent that they can recognize a face even years after a brief encounter [19,20,21,22].

Another crucial cognitive process is spatial navigation, which is fundamental for mobility and independence. This process involves the use of both egocentric and allocentric representations, which enable individuals to perceive distances, recognize landmarks and their spatial relationships, form cognitive maps of the environment (i.e., the ability to mentally represent the surroundings, landmarks and the spatial relation between them [23]), and repeat or retrace previously traveled routes (i.e., route repetition and route retracing) [24,25]. Interestingly, spatial navigation can also be impaired in some individuals due to brain damage (i.e., acquired topographical disorientation [26,27,28]) or impaired from birth (i.e., developmental topographical disorientation [29,30,31,32,33,34]).

At a descriptive level, it seems evident that face recognition and navigational skills are very different cognitive processes. However, some interesting associations between these two skills have been previously reported. Some authors have estimated that approximately 29% of individuals with AP show concurrent navigational deficits [35]. For example, Mr. LL—one of the first documented cases of AP—showed profound deficits not only in recognizing familiar faces, but also in spatial orientation [36,37]. More recent single-case studies have also reported co-occurring deficits in face recognition and navigational skills [38,39,40,41]. Furthermore, a group study of patients with AP showed that while both occipitotemporal and anterior temporal lesions were associated with difficulties in recognizing scenes and houses, only lesions in the former were linked to impairment in forming cognitive maps [42]. Altogether, findings from individuals with AP suggest at least some degree of overlap between the neural systems supporting face recognition and navigation, raising the possibility of shared underlying cognitive mechanisms for these seemingly distinct abilities.

Although some anecdotal reports also suggest similar navigational impairments in DPs [43,44,45], objective evidence is more mixed. For example, an early study examining seven DPs showed that all individuals performed within the normal range in the recognition of scenes and houses and in cognitive map formation [42]. A subsequent study using the Four Mountain Test—a measure of topographical perception and memory [46,47,48]—revealed that four out of eight DPs presented concurrent deficits in topographical memory, but not in topographical perception [49]. A few other single-case studies with individuals diagnosed with either DP or developmental topographical disorientation have reported similar associations between face recognition and navigation deficits [50,51,52,53]. Interestingly, one of these single-case studies presented the case of FN, a DP with concurrent and profound deficits in the formation and retrieval of cognitive maps, who, after a virtual reality rehabilitation program, showed improvement not only in her navigation skills, but also in face recognition [54].

From a theoretical perspective, the reviewed studies directly address one of the oldest debates in visual cognition research: whether the human face recognition system is domain-specific or domain-general. According to the domain-specific hypothesis, face recognition relies on cognitive processes that are specialized for this task (e.g., [55,56]). In contrast, the domain-general hypothesis holds that face recognition depends on general mechanisms that also operate in the processing of non-face objects (e.g., [57,58,59]).

While studies with individuals with AP and DP seem to provide some support for the domain-general hypothesis, there are at least two important issues that cannot be overlooked. First, certain brain areas involved in face recognition—such as the right fusiform face area [60,61]—and in navigation—such as the parahippocampal place area [62,63]—lie in close proximity. Therefore, it is likely that lesions in one of these areas extend to the other, which could explain the concurrent deficits in navigation skills observed in some individuals with AP. Second, studies with DPs have used a different range of tasks to assess navigational and spatial skills, including the Four Mountain Test [49], the cognitive map formation [42] and other in-house navigational tasks [42,50,51,52,53,54]. This not only makes comparisons across studies difficult, but may also contribute to the mixed findings.

More generally, even if we assume the domain-general hypothesis, it remains unclear whether the relationship between face recognition and navigation is merely coincidental (i.e., observable only in specific cases, such as AP and DP) or genuinely interdependent (i.e., such that variation in one ability systematically predicts variation in the other). One way to address this question is to examine navigational skills across the full spectrum of face-recognition ability. Evidence of such an association would not only suggest shared mechanisms but also a genuine interdependence between the two skills.

The present study, therefore, investigates the relationship between face recognition and spatial navigation across the whole spectrum of face recognition skills. To this end, we tested a group of DPs, SRs, and control participants in a recently validated route-learning task designed to evaluate navigational skills in both clinical and non-clinical populations [24,25]. In contrast to other tasks used in previous research, which employed static stimuli [49] or unusual perspectives [42], this new paradigm assesses navigational skills from a first-person perspective and in an environment that more closely resembles real-life settings [24,25]. Another advantage of this task is that it evaluates both the ability to repeat a learned route and to retrace a learned route, which relies on different strategies. To repeat a route, participants can (1) simply memorize a specific sequence of turns (i.e., “left–straight–right–straight”)—although this strategy is prevented in our task by randomizing the intersection at which participants are placed (see Section 2.3); (2) form stimulus–response associations (i.e., “at the intersection with the red building, turn right”); or (3) form sequences of place knowledge, in which participants encode the order in which places are encountered, allowing them to anticipate what comes next [25]. In contrast, route retracing relies more strongly on allocentric strategies, requiring participants to represent—in a view-independent manner—the spatial relationships between where they currently are, where they came from, and where they need to go next [25]. Therefore, compared to previous research, our task not only provides a more comprehensive assessment of spatial navigation but also offers a more ecological measure.

Following the domain-general hypothesis, two possible outcomes would be expected. If face recognition and navigational skills are coincidental but not interdependent, DPs would perform worse than control and SRs, with no differences between the two latter groups. Additionally, if face recognition and navigational skills are interdependent, performance should follow an incremental pattern with SRs showing better performance compared to control and DPs, and controls performing better than DPs. On the contrary, according to the domain-specific hypothesis, we would expect to find no differences in navigational skills across the groups.

## 2. Materials and Methods

### 2.1. Participants

Eighteen adults with DP (15 females, 3 males; mean age = 51.50; SD age = 9.31), 16 SRs (12 females, 4 males; mean age = 46.37; SD age = 6.53), and 28 control participants (14 females, 14 males; mean age = 45.83; SD age = 9.02) completed this study. DPs and SRs had previously contacted us to enquire about their face recognition abilities. To confirm their face recognition deficits, DPs had been assessed with a battery comprising the Cambridge Face Memory Test [64], the Cambridge Face Perception Test [65], and a Famous Face Test [15]. For inclusion as a DP participant, they were required to perform at least 1.7 SDs below published age-matched controls on at least two of the three screening tests. Table 1 presents the DPs’ performance across these tests.

SRs were assessed with the Cambridge Face Memory Test—Extended Form (CFMT+) [66], the Models Memory Test (MMT) [67] and the Pairs Matching Test (PMT) [67]. Individuals were considered to be SRs if they scored at least 1.96 SD above control norms in at least two of the three tests. Table 2 presents SRs performance across these tests.

Note that the assessment criteria used to identify both DPs and SRs align with current standards followed by most researchers in the field [14,67,68,69]. Control participants were recruited through local advertising. All of them scored within the normal range on the CFMT+ (see Table 3). Our sample size is larger than that of other studies exploring the same issue [42,49]. Based on the effect size previously reported by Klargaard and colleagues (d = 1.36) [49], a comparable difference between DPs and controls (partial η^2^ ≈ 0.29) would require a total of approximately 30 participants (i.e., at least 10 per group) to achieve 80% power. For an ANOVA including DPs, controls, and SRs, an effect of similar magnitude across the three groups (partial η^2^ ≈ 0.55) would require only about 12 participants in total (i.e., at least four per group). For the correlation analysis between CFMT scores and performance on the navigation task, detecting a comparable effect (r = 0.56) would require a total of 26 participants. Thus, our study is more than adequately powered relative to previous work. This study was approved by the ethics committee at Bournemouth University (Ethics ID: 45034, approval date: 24 December 2010).

### 2.2. Materials

**Figure 1:**
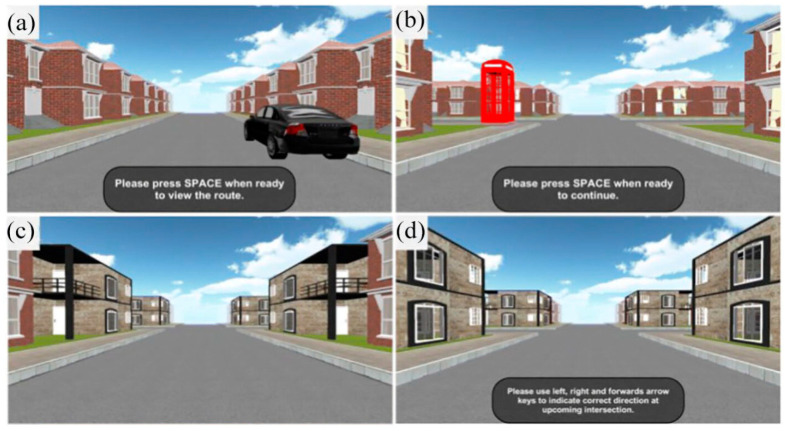
Screenshots showing different points in the learning (upper row) and the repetition phases. (**a**) Start location; (**b**) destination; (**c**,**d**) represent different intersections.

For this study, we adapted the Route Learning and Navigation Test Battery [24]. This battery uses a single route consisting of 12 four-way intersections: 4 straight segments, 4 right turns, and 4 left turns. Along the route, all buildings between intersections were identical, except for the start location, which featured a black car (see Figure 1a), and the destination, which was marked by a red telephone box (see Figure 1b). Each intersection contained a unique landmark building placed in each of the four corners (see Figure 1c,d).

### 2.3. Procedure

Participants were tested in person at the navigation lab at Bournemouth University. The test battery was presented on a computer with a large 42-inch screen. Participants were seated approximately 100 cm from the screen. After signing the consent form, the experiment began.

**Figure 2:**
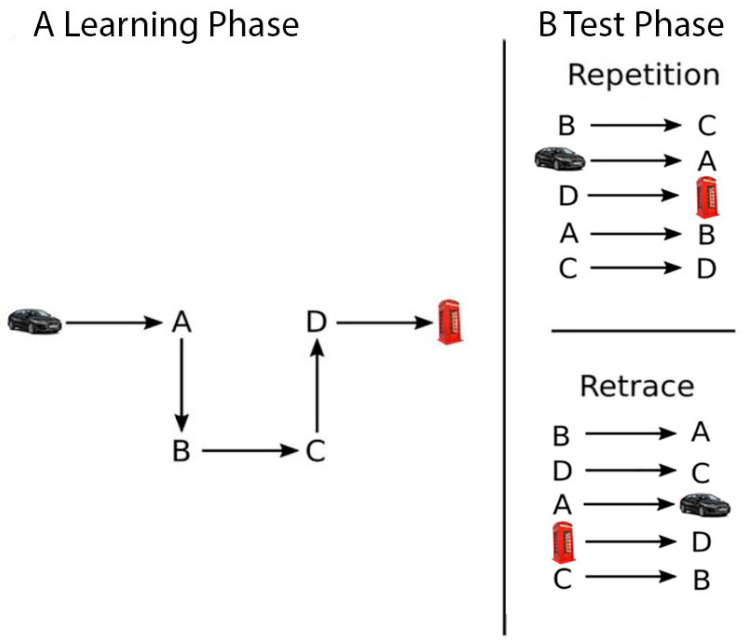
Schematic illustration of the Route Learning and Navigation Test Battery using a shortened route.

There were two different route conditions: the repetition route and the retrace route. The order of the two route conditions was counterbalanced across participants. Each of these conditions had six experimental sessions. The session comprised a learning and a test stage (see Figure 2). In the learning stage (Figure 2A), participants were passively transported along the route from a first-person perspective at a speed of 7.6 m/s and were asked to memorize it. In the test stage (Figure 2B), participants approached each intersection along the route from a first-person perspective and were required to indicate the correct direction to continue (i.e., left, right, or straight) by pressing the corresponding arrow key. The order of intersections was randomized for each participant and each session. This was performed to avoid the use of a memorable sequence of turns required to successfully navigate the learned route [24,70]. In the repetition condition, participants had to navigate the route in the same direction as learned during the learning stage (e.g., from point B to point C; see Figure 2B, top panel). In the retrace condition, participants had to replicate the route in the opposite direction to that learned during the learning stage. To avoid carry-over effects between route conditions, participants learned different routes in the repetition and retracing conditions. Furthermore, to adjust for task difficulty [24,25], the route in the repetition condition was longer, featuring 12 intersections, while the retrace route was shorter, featuring 6 intersections. The dependent variable was the percentage of correct responses.

### 2.4. Data Analysis

All our analyses were conducted using JASP (version 0.95). For each route, we explored group differences with a 2 (Group: Controls, DPs, SRs) × 6 (Experimental session: 1–6) mixed ANOVA. In addition, we conducted Bayesian ANOVAs using JASP’s default priors (fixed-effects r scale = 0.5, random-effects r scale = 1.0, and covariates r scale = 0.354). Bayes Factors (BF_10_ and BF_01_) were computed to quantify the relative evidence for the alternative and null hypotheses, respectively.

To complement group analyses, we also conducted Pearson’s correlation between participants’ scores in the CFMT/CFMT+ and their average performance across sessions in each route condition. Finally, we explored the relationship between face recognition and navigational skills on a case-by-case basis in both DPs and SRs. For this analysis, each DP’s and SR’s average performance across sessions in each of the route conditions was compared to the average performance of controls using modified *t*-tests for single-case analysis [71,72].

## 3. Results

**Figure 3:**
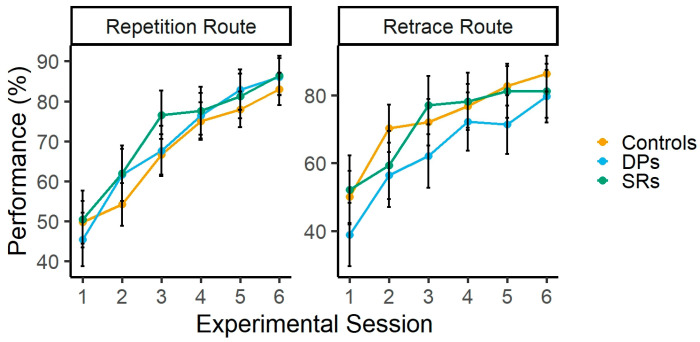
Participants’ performance in the Repetition and Retrace routes. Error bars represent 95% confidence intervals.

Participants’ performance in the repetition and retrace conditions is shown in Figure 3. The ANOVA revealed a main effect of Experimental session [F (5, 295) = 82.41, *p* < 0.001, η^2^_p_ = 0.58], indicating learning effects across experimental sessions. This learning effect was confirmed by a planned linear contrast across the six sessions, *t* (59) = 14.77, *p* < 0.001, with a positive slope estimate of 30.33 (SE = 2.05), suggesting a steady increase in performance over time. Supporting this, the corresponding Bayesian analysis shows that the differences across Experimental sessions are 4.79 × 10^52^ more favored than the lack of differences (BF_10_ = 4.79 × 10^52^). The main effect of group was not significant [F (2, 59) = 0.35, *p* = 0.70, η^2^_p_ = 0.01], showing similar performance in controls (M = 67.75; 95% CI = 61.01 − 74.49), DPs (M = 69.98; 95% CI = 61.57 − 78.39) and SRs (M = 72.39; 95% CI = 63.47 − 81.31). This finding was supported by Bayesian analysis which showed that the lack of differences in performance across groups are 5.54 more favored than the differences across groups (BF_01_ = 5.54). The interaction between these factors also failed to reach statistical significance [F (10, 295) = 1.05, *p =* 0.40, η^2^_p_ = 0.03]. Bayesian analysis showed that the lack of interactions between Group and Experimental session is 22.58 more favored than the interaction (BF_01_ = 22.58).

The same ANOVA for the Retrace route revealed a main effect of Experimental session [F (5, 295) = 34.89, *p <* 0.001, η^2^_p_ = 0.37], suggesting learning effects across experimental sessions. This learning effect was confirmed by a planned linear contrast across the six sessions, *t* (59) = 9.75, *p* < 0.001, with a positive slope estimate of 27.67 (SE = 2.83), suggesting a steady increase in performance over time. This finding was supported by the corresponding Bayesian analysis, which shows that the differences across Experimental sessions are 2.49 × 10^26^ more favored than the lack of differences (BF_10_ = 2.49 × 10^26^). The main effect of group failed to reach statistical significance [F (2, 59) = 1.46, *p =* 0.23, η^2^_p_ = 0.04], which indicates similar performance in controls (M = 73.01; 95% CI = 65.80 − 80.22), DPs (M = 63.42; 95% CI = 54.43 − 72.42) and SRs (M = 71.52; 95% CI = 61.98 − 81.06). This lack of difference across groups was supported by Bayesian analysis, which showed that the lack of differences in performance across groups is 3.19 more favored than the differences across groups (BF_01_ = 3.19). The interaction between Group and Experimental also failed to reach statistical significance [F (10, 295) = 0.82, *p =* 0.60, η^2^_p_ = 0.02]. Bayesian analysis showed that the absence of interaction between these factors is 31.40 more favored than the interaction (BF_01_ = 31.40).

**Figure 4:**
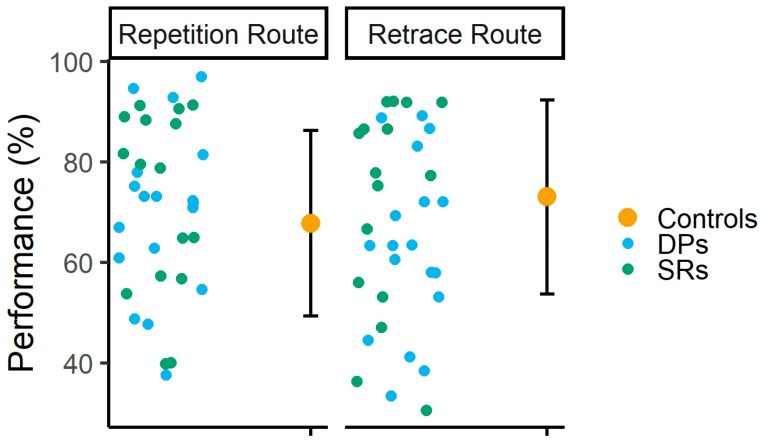
Individual performance of DPs and SRs, along with the average performance of controls. Error bars indicate ±1 standard deviation, a commonly reported measure of dispersion in single-case analyses [71,72].

Pearson correlation analyses revealed no significant correlation between participants’ scores on the CFMT/CFMT+ and their average performance across sessions in the repetition [r (60) = 0.04, *p =* 0.72, 95% CI = −0.20–0.29] and retrace [r (60) = 0.17, *p =* 0.18, 95% CI = −0.08–0.40] routes. Finally, single-case analyses (see Figure 4) revealed that, in the repetition route, none of the DPs or SRs performed significantly differently from the control group. In the retrace route, two DPs performed worse than control participants [both ts (27) ≥ −1.73, *p ≤* 0.04, Z-PCCs ≥ −1.78, 95% CI = −2.35–−1.16]. Interestingly, while none of the SRs performed better than the control participants, two of them did perform worse in this route [both ts (27) ≥ −1.87, *p ≤* 0.03, Z-PCCs ≥ −1.91, 95% CI = −2.53–−1.27].

## 4. Discussion

In this study, we investigated the relationship between face recognition and spatial navigation. Specifically, we examined whether (1) such a relationship exists, as previous research suggests [49,50,54] and—if so—(2) whether this relationship is merely coincidental (i.e., observable only in specific cases, such as AP and DP) or interdependent (i.e., such that variation in one ability systematically predicts variation in the other). DPs, SRs, and control participants were tested with a recently validated route-learning task that evaluates both participants’ ability to repeat a learned route (i.e., route repetition) and their ability to return to the starting point of the learned route (i.e., route retracing) [24]. In both route conditions, we found a clear linear effect of learning, replicating previous findings with this task [24,25]. Our group analysis showed that performance in both route repetition and route retracing was comparable among DPs, SRs, and control participants. These results were supported by our individual analysis, which showed that none of the DPs or SRs differed significantly from the control group in the repetition route condition, whereas two DPs and two SRs performed worse than control participants in the retrace route condition. Altogether, our results suggest that face recognition and navigational skills are not associated, challenging the domain-general hypothesis and supporting the domain-specific hypothesis.

While previous studies have reported clear concurrent navigational deficits in AP after occipitotemporal lesions [42], such deficits are less clear in DP. For example, Corrow and colleagues [42] found that DPs’ performance in cognitive map formation was comparable to that of control participants. Klargaad and colleagues [49] reported that, at a group level, DPs were impaired in topographical memory as measured with the Four Mountain Test. However, individual analysis revealed that only 4 out of 8 DPs were impaired when compared to the control group. Our results, with a larger sample of DPs alongside control participants and SRs, suggest neither a coincidental nor an interdependent relationship between face recognition and navigational skills. It could be argued that our results might reflect a lack of power to detect group differences. This is, however, unlikely, as our study is better powered than previous work [42,49]. In addition, our group-level findings were supported by single-case analyses, which were conducted using a lenient one-tailed criterion to maximize sensitivity to genuine differences.

Thus, in light of our results, one question that arises is why other studies have reported an association between face recognition and navigational skills specifically in the lower end of the face recognition distribution. In the case of AP, it is possible that instead of shared cognitive resources, such deficits can be explained by widespread lesions affecting both face recognition and navigation. This is supported by the fact that only those APs with occipitotemporal lesions—but not those with anterior temporal lesions—show concurrent navigational deficits [42].

While valid for AP, this reasoning, however, does not explain why some DPs also present concurrent navigational deficits, as their impairments are not circumscribed to brain lesions [16,73]. One important difference between our study and others reporting navigational deficits in DPs lies in the assessment criteria used to define DP. In our case, participants were evaluated with the CFMT, the CFPT, and the FFT, and only those showing impaired performance on at least two of these three tasks were included. However, other studies reporting concurrent navigation deficits in DP have used more relaxed inclusion criteria, such as impaired performance on the CFMT alone [48] (but see [53]). Therefore, it is possible that differences in the inclusion criteria of DPs might explain the inconsistent findings.

Another important difference across studies is the task used to evaluate navigational skills. In this study, we employed an ecological route-learning task that simulates a real-life route-learning scenario and has been previously validated to assess navigational skills in both clinical and non-clinical populations [24,25]. This task requires participants to associate specific landmarks with directions (i.e., turn left at the intersection with the building with red bricks). However, the task may not capture subtler aspects of navigation that could underlie previously reported associations between face recognition and navigational skills. For instance, the only group study reporting such an association employed the Four Mountain Test [49]. In this test, participants are required to recognize a landscape consisting of four mountains from a different perspective than the one in which it was learned [46,47,48]. While this test has been criticized for its limited ecological validity and the strong demand on non-navigational cognitive processes, such as attention and working memory [74,75], it is thought to reflect participants’ ability to represent metric relationships within space [27,49], a process that could be more akin to the so-called configural processing of faces, which, according to some authors, is the signature of face recognition [76,77]. This potential relationship between the ability to process metric distances within space and face recognition could be explored in future research by testing DPs, SRs, and control participants with navigational tasks that require the use of such metric information, such as the Four Mountain Test or other spatial configuration tasks (see, e.g., [47]). Conversely, this relationship can also be examined by comparing patients with topographical disorientation [26,27,28,29,30,31,32,33,34] and control participants in tasks requiring the discrimination of changes in the configural information of faces [76].

## 5. Conclusions

This study aimed to explore the relationship between navigational skills and face recognition. Using an ecological measure of spatial navigation that assesses, from a first-person perspective, both the ability to repeat and retrace a route, our results showed no differences in navigational skills between DPs, SRs, and control participants. These findings challenge previous claims of an association between face recognition and spatial navigation and instead support the domain-specific hypothesis of face recognition.

## Figures and Tables

**Table 1:** DPs’ performance on the Cambridge Face Memory Test (CFMT), Cambridge Face Perception Test (CFPT), and the Famous Faces Test (FFT).

ID	Age	Sex	CFMT (Total = 72; Cutoff = 46)	CFPT (No. Errors; Cutoff = 58)	FFT (% Correct; Cutoff = 80.00%)
ANDP01	62	F	44	78	75
ANDP02	60	F	41	62	75
ANDP03	60	F	33	54	55.93
ANDP04	59	M	34	54	56
ANDP08	57	F	42	66	21.05
ANDP09	58	F	42	38	71.67
ANDP10	45	F	36	44	75
ANDP11	58	M	39	64	36
ANDP12	45	F	39	38	69.23
ANDP13	54	F	38	38	75
ANDP14	31	F	34	64	80
ANDP15	45	F	41	66	80.36
ANDP16	36	F	40	52	47.37
ANDP17	58	F	45	58	42.86
ANDP18	57	F	46	56	28.57
ANDP19	46	F	42	48	50
ANDP22	56	F	32	80	32.14
ANDP23	40	M	38	42	68

**Table 2:** SRs’ performance on the Cambridge Face Memory Test—Extended Form (CFMT+), the Models Memory Test (MMT), and the Pairs Matching Test (PMT).

ID	Age	Sex	CFMT+ (Total = 102; Cutoff = 90)	MMT (Total = 90; Cutoff = 73)	PMT (Total = 48; Cutoff = 40)
ANSR01	37	F	93	81	40
ANSR02	42	F	94	79	37
ANSR03	41	F	95	74	37
ANSR05	59	F	94	80	35
ANSR07	51	F	96	66	41
ANSR08	43	F	91	83	42
ANSR10	53	F	88	76	43
ANSR12	36	F	94	74	37
ANSR13	45	M	94	87	43
ANSR14	45	F	93	88	43
ANSR101	41	F	92	71	45
ANSR102	47	M	90	82	37
ANSR103	47	M	97	80	35
ANSR104	49	F	92	77	39
ANSR105	49	F	98	78	41
ANSR106	57	M	97	79	43

**Table 3:** Controls’ performance on the CFMT+.

ID	Age	Sex	CFMT+ (Total = 102)
ANC01	44	F	66
ANC02	52	F	62
ANC04	54	F	62
ANC05	55	F	85
ANC06	38	F	81
ANC07	42	F	85
ANC09	38	M	80
ANC10	34	M	65
ANC11	45	F	69
ANC12	41	F	59
ANC14	45	M	75
ANC16	32	M	59
ANC19	43	M	68
ANC20	61	F	71
ANC22	43	F	74
ANC23	56	M	55
ANC26	32	M	71
ANC27	37	F	81
ANC30	55	M	84
ANC31	50	F	58
ANC32	39	F	88
ANC33	59	F	68
ANC35	40	M	69
ANC36	55	M	65
ANC37	36	M	78
ANC39	50	F	81
ANC40	60	M	72
ANC41	55	M	57
ANC42	38	M	60
ANC01	44	F	66
ANC02	52	F	62
ANC04	54	F	62

## Data Availability

The data supporting this study can be downloaded at https://osf.io/z4g8j/.

## Abbreviations

The following abbreviations are used in this manuscript:

AP	Acquired Prosopagnosia
DP	Developmental Prosopagnosia
SR	Super-Recognizer
CFMT	Cambridge Face Memory Test
CFMT+	Cambridge Face Memory Test—Extended Form
CFPT	Cambridge Face Perception Test
MMT	Models Memory Test
PMT	Pairs Matching Test
FFT	Famous Faces Test
